# Reducing error: benign abnormalities mimicking malignancy when reporting scans of patients with known malignancy

**DOI:** 10.1186/1470-7330-15-S1-P29

**Published:** 2015-10-02

**Authors:** S Jenkins, G Joseph

**Affiliations:** 1 Velindre Cancer Centre, Cardiff, UK

## Learning objectives

To improve the ability to accurately report CT and MRI by learning to recognise benign findings that may mimic malignant pathology in the setting of scans performed on patients with known malignancy.

## Content organisation

CT and MR images will be displayed demonstrating benign abnormalities that are easily misinterpreted as metastases, recurrent tumour or residual tumour in patients with known malignancy. Examples of benign findings will be displayed next to the corresponding malignant abnormalities to demonstrate the potential error.

## Conclusion

The ability to accurately differentiate between benign and malignant disease can be challenging and can contribute to reporting discrepancies even amongst experienced radiologists. This can be especially demanding when imaging patients with known malignancy and is a concern for general and specialist radiologists. Being aware of abnormalities that mimic malignancy will help to increase the accuracy of CT and MR reports and ensure the correct treatment plans are implemented.

